# Traumatic axonal injury influences the cognitive effect of non-invasive brain stimulation

**DOI:** 10.1093/brain/awz252

**Published:** 2019-08-30

**Authors:** Lucia M Li, Ines R Violante, Karl Zimmerman, Rob Leech, Adam Hampshire, Maneesh Patel, Alexander Opitz, David McArthur, Amy Jolly, David W Carmichael, David J Sharp

**Affiliations:** 1 Computational, Cognitive and Clinical Imaging Lab, Division of Brain Sciences, Department of Medicine, Imperial College London, UK; 2 UK DRI Centre for Care Research and Technology, Imperial College London, UK; 3 School of Psychology, Faculty of Health and Medical Sciences, University of Surrey, UK; 4 Centre of Neuroimaging Science, Kings College London, UK; 5 Department of Imaging, Charing Cross Hospital, London, UK; 6 Department of Biomedical Engineering, University of Minnesota, Minneapolis, MN, USA; 7 David Geffen School of Medicine, UCLA, Los Angeles, CA, USA; 8 Biomedical Engineering Department, Kings College London, UK

**Keywords:** brain stimulation, response inhibition, traumatic brain injury, salience network, axonal injury

## Abstract

Non-invasive brain stimulation has been widely investigated as a potential treatment for a range of neurological and psychiatric conditions, including brain injury. However, the behavioural effects of brain stimulation are variable, for reasons that are poorly understood. This is a particular challenge for traumatic brain injury, where patterns of damage and their clinical effects are heterogeneous. Here we test the hypothesis that the response to transcranial direct current stimulation following traumatic brain injury is dependent on white matter damage within the stimulated network. We used a novel simultaneous stimulation-MRI protocol applying anodal, cathodal and sham stimulation to 24 healthy control subjects and 35 patients with moderate/severe traumatic brain injury. Stimulation was applied to the right inferior frontal gyrus/anterior insula node of the salience network, which was targeted because our previous work had shown its importance to executive function. Stimulation was applied during performance of the Stop Signal Task, which assesses response inhibition, a key component of executive function. Structural MRI was used to assess the extent of brain injury, including diffusion MRI assessment of post-traumatic axonal injury. Functional MRI, which was simultaneously acquired to delivery of stimulation, assessed the effects of stimulation on cognitive network function. Anodal stimulation improved response inhibition in control participants, an effect that was not observed in the patient group. The extent of traumatic axonal injury within the salience network strongly influenced the behavioural response to stimulation. Increasing damage to the tract connecting the stimulated right inferior frontal gyrus/anterior insula to the rest of the salience network was associated with reduced beneficial effects of stimulation. In addition, anodal stimulation normalized default mode network activation in patients with poor response inhibition, suggesting that stimulation modulates communication between the networks involved in supporting cognitive control. These results demonstrate an important principle: that white matter structure of the connections within a stimulated brain network influences the behavioural response to stimulation. This suggests that a personalized approach to non-invasive brain stimulation is likely to be necessary, with structural integrity of the targeted brain networks an important criterion for patient selection and an individualized approach to the selection of stimulation parameters.

## Teaser

The behavioural effects of non-invasive brain stimulation are highly variable. Li *et al.* show that the effects of transcranial direct current stimulation on cognitive function after traumatic brain injury are heavily influenced by white matter damage within the stimulated network. A personalized approach to selecting stimulation parameters may therefore be required.

## Introduction

Non-invasive brain stimulation techniques have been widely used to try to improve function after brain injury (Liew *et al.*, 2014; Li *et al.*, 2015). Transcranial direct current stimulation (TDCS), which modulates neuronal excitability by delivering low electrical currents to the brain through scalp electrodes, has been of particular interest because of its relative ease of use and good safety profile (Purpura and Mcmurtry, 1965; Brunoni *et al.*, 2011; Stagg and Nitsche 2011). TDCS has shown potential to improve cognitive function in both healthy controls and a range of neurological conditions (Kuo and Nitsche, 2012). However, the behavioural effects are highly variable, for reasons that are poorly understood. Variability in response to stimulation is a particular issue when using TDCS for cognitive enhancement after brain injury because injury heterogeneity is characteristic of many of the conditions where brain stimulation is being trialled. Here we test whether the response to TDCS following traumatic brain injury (TBI) is dependent on the structure of the brain networks stimulated.

TBI commonly causes long-term deficits in cognitive control. Response inhibition is an important aspect of cognitive control, a process that we have previously investigated in TBI using the Stop Signal Task (SST). In this task, participants are required to inhibit a response to an unexpected and behaviourally salient stimulus, requiring a switch from automatic to controlled behaviour (Hampshire and Sharp, 2015). During this response inhibition, the brain’s activity is characterized by salience network activation and concurrent default mode network deactivation (Sharp *et al.*, 2010). This pattern of anti-correlated activity is related to task performance, with greater salience network activation and default mode network deactivation associated with better performance (Congdon *et al.*, 2011). Additionally, a lack of default mode network deactivation and reduced functional connectivity between the salience and default mode networks in patients with TBI is associated with poor SST performance (Bonnelle *et al.*, 2012; Jilka *et al.*, 2014).

The right inferior frontal/anterior insula node (rIFG/AI) of the salience network is thought to play a specific role in mediating a switch between different cognitive states and the associated anti-correlation of salience and default mode networks activity (Mesulam, 1990; Sridharan *et al.*, 2008; Hampshire and Sharp, 2015). Traumatic axonal injury is an important consequence of TBI, and can arise from chronic processes such as inflammation as well as the diffuse axonal injury is sustained from shearing forces at time of injury (Johnson *et al.*, 2013; Hill *et al.*, 2016). This axonal injury results in damage to white matter tracts connecting anatomically distributed brain regions, and relates to cognitive deficits (Kinnunen *et al.*, 2011). Specifically, post-TBI damage within the white matter tract linking the two nodes of the salience network [the rAI and the dorsal anterior cingulate (dACC)/pre-supplementary motor area (pre-SMA)] is associated with reduced functional connectivity between the salience and default mode networks, abnormal default mode network activation, and impaired response inhibition (Bonnelle *et al.*, 2012; Jilka *et al.*, 2014). Therefore, abnormal salience and default mode network function is a signature of cognitive deficit after TBI, and white matter damage within the salience network appears to be an important contributor to this dysfunction (Sharp *et al.*, 2014). This suggests that the salience network is a potential target for normalizing cognitive network dysfunction and improving cognition post-TBI.

We have previously shown that TDCS targeted to the rIFG/AI node of the salience network in healthy controls modulates both salience and default mode network activity (Li *et al.*, 2018). Additionally, TDCS has shown potential for cognitive enhancement in four TBI studies (Kang *et al.*, 2012; Leśniak *et al.*, 2014; Sacco *et al.*, 2016; O’Neil-Pirozzi *et al.*, 2017). However, these studies report variable behavioural outcomes, and did not explore the interindividual factors that may underlie variability in response to TDCS. White matter damage in the TBI population is widespread but there is also great heterogeneity in its distribution and severity of white matter injury (Presson *et al.*, 2015). Given the important contribution of white matter injury to post-TBI cognitive deficits and abnormal network function, variability in integrity of white matter connections may explain variability in behavioural response to stimulation.

In this study, we used a novel combined TDCS-functional MRI experiment to investigate the effect of salience network stimulation on cognitive networks and response inhibition in moderate-severe TBI patients in the chronic phase of recovery. We also acquired diffusion MRI to investigate whether white matter structure within the salience network would influence the behavioural response to stimulation. We delivered anodal and cathodal TDCS to the rIFG/AI node of the salience network of patients during SST performance, and simultaneously acquired functional MRI. We tested the hypotheses that: (i) anodal rIFG/AI stimulation can improve SST performance; (ii) white matter integrity within the salience network influences the behavioural response to TDCS; and (iii) anodal rIFG/AI stimulation improves the previously observed abnormal salience and default mode network function after TBI (Bonnelle *et al.*, 2012; Jilka *et al.*, 2014).

## Materials and methods

### Participants

Patients with moderate-severe TBI (*n = *35, five female, 30 male) were recruited, based on Mayo classification (Malec *et al.*, 2007). Inclusion criteria were: 16–80 years old, ≥6 months post-injury. Exclusion criteria included: contraindications to MRI or TDCS, significant premorbid neurological or psychiatric history. Further group characteristics are presented in Supplementary Table 1. The mean age was 39.6 years old [standard deviation (SD) 10.1 years, range 21–56]. The average time since injury was 48.9 months (SD 95.6 months, range 6.5–367 months). Four TBI participants were excluded from analyses: one because he was found to have a large cortical lesion over the site of stimulation; two failed performance criteria on the SST; and one further participant’s structural imaging showed extensive white matter damage within the salience network tract (Supplementary Fig. 1). A cohort of healthy control participants also completed the study (*n = *24, 12 female, 12 male, mean age 39 years, SD 15.8 years). Participants had no history of psychiatric or neurological illness, previous TBI, alcohol or substance misuse. All participants provided written informed consent and were naïve to TDCS. The study conforms to the Declaration of Helsinki and ethical approval was granted through the local ethics board [National Research Ethics Service (NRES) Committee London – West London and GTAC].

Compared to controls, 19 TBI participants were classified as impaired using a multivariate normative comparison (MNC) approach (Huizenga *et al.*, 2007) on the following tests: Trail Making, delayed recall and discrimination scores on the Hopkins Verbal Learning Test-Revised (Brandt and Benedict, 2001) and Brief Visuospatial Learning test-revised (Benedict *et al.*, 1998) and the colour-word Stroop task (Delis *et al.*, 2001).

### Stop Signal Task

**Figure 1:**
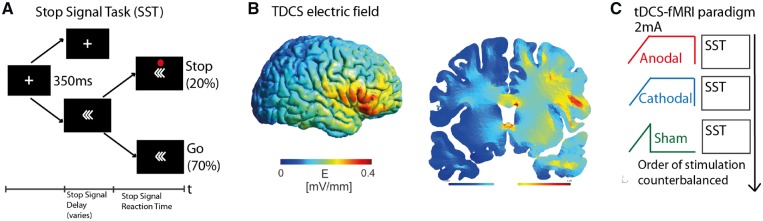
**Materials and methods.** (**A**) Stimuli in the SST. (**B**) Current density model based on the montage used showing maximum electric field strength over the right inferior frontal region. Modelling is based on non-injured standard brain and tissue. (**C**) The TDCS/functional MRI paradigm, comprising three separate runs of the SST with concurrent TDCS (anodal, cathodal and sham), with a 2–3-min break between each run.

The SST used in this study has been described previously (Bonnelle *et al.*, 2012). In brief, participants are instructed to press a button held in their left or right hand in response to left or right pointing arrows, respectively. Infrequently, a red dot, the ‘stop’ signal, appeared above the arrow after a variable interval. Participants had to withhold their button press in response to the ‘stop’ signal. There were 184 trials in total, comprising 20% ‘stop’ trials, 70% normal (‘go’) trials and 10% rest trials (Fig. 1A). To minimize tactical waiting for the appearance of the ‘stop’ signal, a negative feedback screen saying ‘Speed Up’ was presented if slowing reaction times were detected. The main outcome measure is the stop signal reaction time (SSRT). It is a composite measure that accounts for an individual’s motor reaction time. It is calculated as [mean reaction time − stop signal delay], where stop signal delay is the interval between presentation of the arrows and appearance of the red dot ‘stop’ signal that produced successful stopping on 50% of ‘stop’ trials. A low SSRT indicates good task performance, indicating that little time is required to countermand the prepared motor response. Behavioural response to TDCS was defined as the difference in SSRT between active and sham TDCS (ΔSSRT_anodal_ and ΔSSRT_cathodal_). A more negative value indicates greater improvement in performance under active TDCS.

### Transcranial direct current stimulation

TDCS was delivered concurrently to SST task performance and functional MRI acquisition using a magnetic resonance-compatible battery-driven stimulator (NeuroConn), with a previously described circuit (Violante *et al.*, 2017). The ‘active’ electrode (4.5-cm diameter circular rubber electrode) was placed over F8 (based on the 10–20 EEG International system), corresponding to the pars triangularis of the rIFG, and the ‘return’ electrode (7 × 5 cm rectangular rubber electrode) was on the right shoulder with its longitudinal axis parallel to the coronal plane (centre of electrode placed over midpoint between tip of the acromion and base of neck). Numerical modelling showed maximal current density over the right inferior frontal region (Fig. 1B). Anodal and cathodal TDCS were delivered at 2 mA (for full parameters see Supplementary material).

### MRI acquisition

T_1_, FLAIR, susceptibility-weighted imaging (SWI) or T_2_* gradient echo, functional MRI and diffusion tensor imaging (DTI) sequences for all participants were acquired on a 3 T Siemens Verio (Siemens), using a 32-channel head coil. Structural scans were inspected by a consultant neuroradiologist (see Supplementary material for full parameters and further information).

#### Functional MRI acquisition and analysis

Functional MRI was acquired while participants performed the SST in an event-related design, the details of which have been described previously (Sharp *et al.*, 2010). Each participant performed three runs of the SSRT, under sham, anodal and cathodal TDCS. The runs were sequential, with a brief break in between each run during which the participant remained in the MRI scanner. The order was counterbalanced between participants to minimize risk of systematic bias from any potential carryover effects (Fig. 1C).

All functional MRI data were preprocessed and analysed using the FMRIB software suite. Full details are in the Supplementary material. Briefly, functional MRI images were brain extracted, motion corrected and registered to standard space in FMRIB’s Software Library (FSL; Smith *et al.*, 2004; Jenkinson *et al.*, 2012). FMRIB’s ICA-based Xnoiseifier (FIX; Griffanti *et al.*, 2014; Salimi-Khorshidi *et al.*, 2014) was used to remove noise components further.

The functional MRI-TDCS SST was analysed with FSL’s FMRI Expert Analysis Tool (FEAT) (Smith *et al.*, 2004; Jenkinson *et al.*, 2012). To address heterogeneity within the TBI group, as in previous studies, we median-split the TBI participants into two groups based on SST performance in the baseline (sham) condition (‘poor’ and ‘good’ performers). A higher-level mixed effects analysis was performed to determine differences in activation between these two groups for each stimulation condition. A separate higher-level mixed effects analysis was run to directly compare stimulation conditions, using the ‘Triple T-test’ GLM set-up within FSL FEAT. The final Z statistical images were thresholded using a Gaussian random field-based cluster inference with a height determined by a threshold of z > 3.1 and a corrected cluster significance threshold of *P = *0.05.

We investigated the effect of TDCS on salience network and default mode network functional connectivity with generalized psychophysiological interaction (PPI) analyses (Friston *et al.*, 1997; O’Reilly *et al.*, 2012). We used the following seed regions of interest: rIFG, rAI, dACC/pre-SMA (forming the salience network); ventromedial prefrontal cortex, and the ventral and posterior cingulate cortices (forming the default mode network). A separate group of control participants was used to define the regions of interest. Whole brain PPI was carried out to determine the functional connectivity between the region of interest and the whole brain.

#### Diffusion tensor imaging acquisition and analysis

White matter integrity was assessed with DTI, performed as the final scan in the session, with acquisition parameters as described (Supplementary material). Diffusion data were preprocessed within FSL and DTITK to build individual fractional anisotropy maps (Smith, 2002; Zhang *et al.*, 2006, 2010).

A region of interest approach was used to assess white matter structural connectivity in the salience and default mode networks. Fractional anisotropy values were extracted from each participant from the following previously described tracts (Bonnelle *et al.*, 2012):

- rAI-dACC/pre-SMA tract (to assess salience network structural integrity in the tract connecting the rAI to the dACC/pre-SMA). This tract partly overlaps the frontal aslant tract described by Catani *et al.* (2011).
- mPFC-PCC/PRE (medial prefrontal cortex to posterior cingulate cortex/precuneus) tract (to assess default mode network structural integrity within the cingulum).

See Supplementary material for full details of DTI preprocessing and analyses.

### Statistical analysis of behavioural results

Statistical analyses of task performance were conducted using MATLAB (R2017a Mathworks, Natick, MA) and R (v.3.4.3 www.r-project.org). ANOVAs with two factors, Stimulation (three levels: sham, anodal, cathodal) and Group (two levels: TBI and controls) were performed for each behavioural measure. We also constructed linear mixed effects models to investigate individual and injury factors that might be associated with behavioural response to TDCS. One model investigated factors associated with the behavioural response to stimulation in both patients and controls (combined model), and the other investigated factors associated with the behavioural response to TDCS in TBI participants only (TBI only). The dependent variable was the change in SSRT under active TDCS compared to sham TDCS (ΔSSRT), a negative value indicating improvement. The following factors were investigated in both models: age, fractional anisotropy within the rAI-dACC/pre-SMA tract, fractional anisotropy within the cingulum tract (mPFC-PCC/PRE), default mode network activation during response inhibition, rAI-default mode network functional connectivity during response inhibition. The following additional factors were investigated in the TBI only model: time since injury (in months), % of lesion volume, whether patients had a significantly impaired MNC score (binary classification). Stimulation type (anodal/cathodal) was also included as a variable. See Supplementary material for full details of model construction.

### Data availability

The authors confirm that the data supporting the findings of this study are available within the article and its Supplementary material. Raw data that support the findings of this study are available from the corresponding author, upon reasonable request.

## Results

### Behavioural analyses

#### TBI patients have greater performance variability than controls

The SSRT is the main outcome measure of the SST. The SSRT is a composite measure, and calculated as [mean reaction time − stop signal delay], where stop signal delay is the interval between presentation of the arrows and appearance of the red dot ‘stop’ signal, which produced successful stopping on 50% of ‘stop’ trials. A longer stop signal delay and a shorter SSRT indicates better SST performance. The baseline task performance (SSRT under sham) of TBI participants, as a group, was not significantly worse than controls. However, there was greater variability in the performance of TBI participants (TBI: mean SSRT = 328.1 ms, SD = 79.8 ms; Control: mean SSRT = 321.7 ms, SD = 48.0 ms) (Table 1), and TBI patient performance also had a bimodal distribution (Supplementary Fig. 2). Heterogeneity in TBI populations is commonly observed, and we have found this to be the case in previous studies (Bonnelle *et al.*, 2011). As previously, for the neuroimaging analyses, we split our TBI participants into two groups: good and poor performers, based on the baseline SST performance.

**Table 1:** Behavioural measures for the Stop Signal Task

	Anodal	Cathodal	Sham
*Controls*			
SSRT (ms)	291.3 ± 58.4	295.1 ± 58.6	321.7 ± 48.0
Stop Signal Delay (ms)	253.7 ± 159.3	230.8 ± 105.7	200.8 ± 94.9
Incorrect stop RT (ms)	523.0 ± 104.7	495.8 ± 71.5	496.1 ± 75.1
Mean RT (ms)	545.1 ± 121.1	525.9 ± 87.6	522.6 ± 89.4
*TBI*			
SSRT (ms)	343.4 ± 73.0	326.5 ± 62.6	328.1 ± 79.8
Stop Signal Delay (ms)	216.9 ± 88.3	234.0 ± 95.0	235.1 ± 131.1
Incorrect stop RT (ms)	539.7 ± 78.5	544.1 ± 67.0	541.6 ± 77.0
Mean RT (ms)	575.1 ± 86.2	577.5 ± 76.8	578.6 ± 83.1

Values represent mean ± standard deviation. RT = reaction time.

#### Anodal TDCS improved response inhibition in control, but not TBI participants

**Figure 2:**
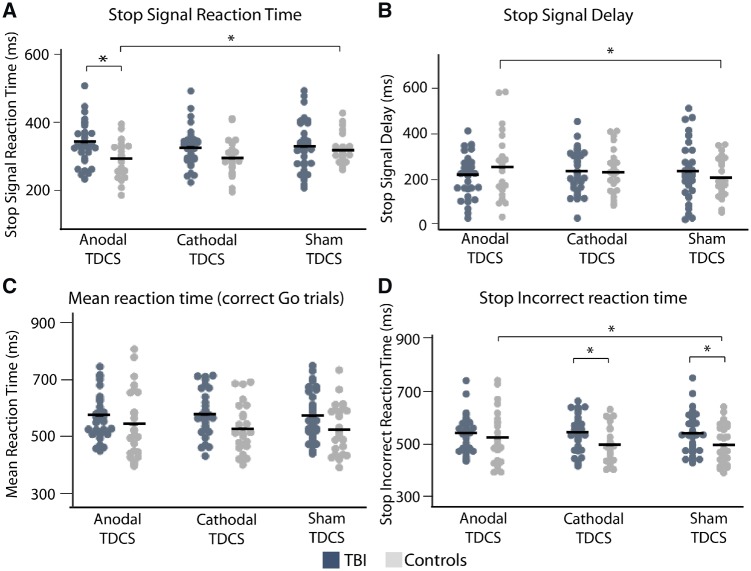
**SST performance with TDCS.** The (**A**) SSRT, (**B**) stop signal delay, (**C**) mean reaction time and (**D**) stop incorrect reaction time for TBI (dark grey) and control (light grey) participants under sham, anodal and cathodal TDCS. Black lines are group mean values.

There was a Group (TBI or controls) × Stimulation type (sham, anodal or cathodal) interaction [*F*(2,110) = 3.94, *P = *0.022] (Fig. 2A). *Post hoc t*-tests showed this to be characterized by a significant improvement in SSRT with anodal TDCS in control but not in TBI participants [*t*(23) = 2.17, *P = *0.041], and a subthreshold improvement in SSRT with cathodal TDCS in controls [*t*(23) = 1.87, *P = *0.074]. Mean improvement in control participants was 30.4 ms (SD 68.7 ms) with anodal and 26.7 ms (SD 69.9 ms) with cathodal TDCS. In TBI, mean SSRT worsened by 21.5 ms (SD 80.4 ms) with anodal TDCS and was almost unchanged (0.0005 ms, SD 60.2 ms) by cathodal TDCS. Control participants also had a significantly lower SSRT under anodal TDCS, compared with TBI participants [anodal: *t*(52.5) = 2.75, *P = *0.008] (Table 1).

There was also a Group × Stimulation type interaction on the stop signal delay [*F*(2,110) = 3.97, *P = *0.022] (Fig. 2B). *Post hoc t*-tests showed this to be due to a significant increase in stop signal delay, indicating improved response inhibition, with anodal compared to sham TDCS in control, but not in TBI, participants [control: *t*(23) = 2.53, *P = *0.019; TBI: *t*(30) = 1.1, *P = *0.291].

The stop incorrect reaction time (SIRT) is the reaction time when participants fail to inhibit a response to a stop signal. A Group × Stimulation type interaction was present for the SIRT [*F*(2,110) = 2.12, *P = *0.048] (Fig. 2D). *Post hoc t*-tests showed this to be characterized by a significant increase in SIRT under anodal compared to sham TDCS in controls, but not in TBI patients [control: *t*(23) = 2.32, *P = *0.029; TBI: *t*(30) = 0.67, *P = *0.498]. There was also a significant difference in the SIRT between control and TBI participants under sham and cathodal TDCS [cathodal: *t*(49) = 2.23, *P = *0.030; sham: *t*(51) = 2.00, *P = *0.050]. There were no main effects or interactions for mean reaction time (all *F* < 2, *P* > 0.05) (Fig. 2C), suggesting that the effects of TDCS were not simply produced by effects on general motor speed.

#### Behavioural response to anodal TDCS strongly correlates with salience network white matter integrity

**Figure 3:**
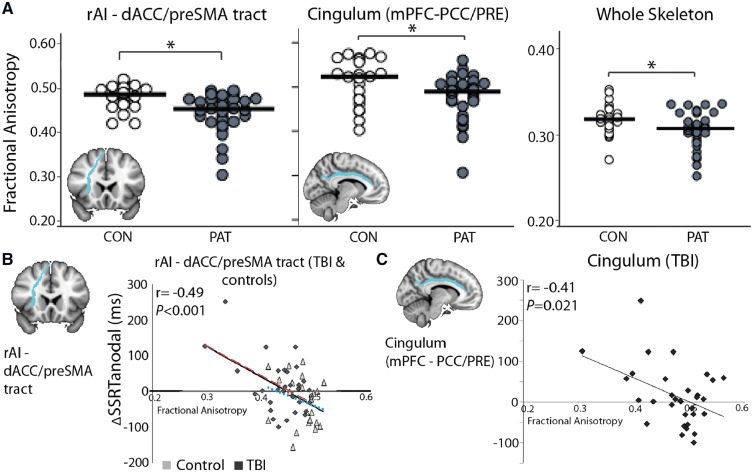
**Relationship between behavioural response to anodal TDCS and white matter integrity.** (**A**) White matter integrity, assessed as fractional anisotropy, of the rAI-dACC/pre-SMA tract, cingulum and whole skeleton in TBI and control participants. Black lines are group mean values. (**B**) Correlation between rAI-dACC/pre-SMA tract integrity and ΔSSRT_anodal_ across both control (light grey) and TBI (dark) participants, significant after Bonferroni correction for multiple comparisons. Black trend line represents correlation across all participants; red dashed trend line represents correlation within TBI participants only; blue dotted trend line represents correlation within control participants only. (**C**) Correlation between cingulum white matter integrity (fractional anisotropy) and ΔSSRT_anodal_ within TBI participants. *Inset*: B brain pictures show the rAI-dACC/pre-SMA tract and the cingulum.

We investigated whether white matter integrity could explain the absence of behavioural effects of TDCS in TBI patients. We have shown that white matter damage within the salience and default mode networks after TBI is associated with worse performance on the SST and more general impairments of cognitive control and attention (Bonnelle *et al.*, 2011, 2012; Jilka *et al.*, 2014). Therefore, we investigated whether salience and default mode network white matter integrity also influenced the behavioural response to TDCS. TBI participants had reduced mean fractional anisotropy within the salience network, in the rAI-dACC/pre-SMA tract [*t*(50.5) = 3.65, *P < *0.001], and default mode network, in the cingulum bundle [*t*(30.2) = 2.81, *P = *0.009], in agreement with previous findings (Bonnelle *et al.*, 2012) (Fig. 3A, inset). Across all participants (TBI and controls), structural integrity measured by fractional anisotropy in the rAI-dACC/pre-SMA tract was negatively associated with the change in SSRT during anodal stimulation (ΔSSRT_anodal_), i.e. higher fractional anisotropy was associated with greater behavioural improvement (r = −0.49, *P < *0.001) (Fig. 3B, right). This effect was significant after Bonferroni correction for multiple comparisons. Two patients had microhaemorrhages (seen on SWI only) within the region of the rAI-dACC/pre-SMA tract and three patients had lesions that had a small degree of lesion overlap with the rIFG and rAI. With the exclusion of these patients, the relationship between (ΔSSRT_anodal_) and rAI-dACC/pre-SMA fractional anisotropy was still strongly significant (r = −0.47, *P < *0.001).

There was no interaction between group (TBI or control) and rAI-dACC/pre-SMA tract fractional anisotropy. For cingulum fractional anisotropy, there was an interaction between group and fractional anisotropy [*F*(1,50) = 5.38, *P = *0.025]. In patients, cingulum fractional anisotropy was negatively correlated with ΔSSRT_anodal_; i.e. higher fractional anisotropy related to better performance under anodal TDCS (r = −0.41, *P = *0.021), though this did not survive multiple comparisons correction (Fig. 3C). This effect was not seen in controls.

Fractional anisotropy within the rAI-dACC/pre-SMA and cingulum tracts were also negatively correlated with the effect of cathodal stimulation on SSRT within patients (rAI-dACC/pre-SMA tract: r = −0.38, *P = *0.034; cingulum: r = −0.39, *P = *0.031) (Supplementary Fig. 3). However, these correlations did not survive correction for multiple comparisons. There was no correlation between whole skeleton fractional anisotropy or lesion volume % and either ΔSSRT_cathodal_ or ΔSSRT_anodal_.

### Neuroimaging: network activity

#### Abnormal default mode network deactivation in impaired TBI patients is related to salience network structural connectivity

Previously, we showed that TBI patients with poor sustained attention and SST performance have abnormal default mode network activation, and that the integrity of the rAI-dACC/pre-SMA tract correlates with this abnormal default mode network activation (Bonnelle *et al.*, 2011, 2012). We investigated whether this finding was also present in this group of TBI patients. As previously, we divided the patients into good and poor performers based on baseline SST performance.

**Figure 4:**
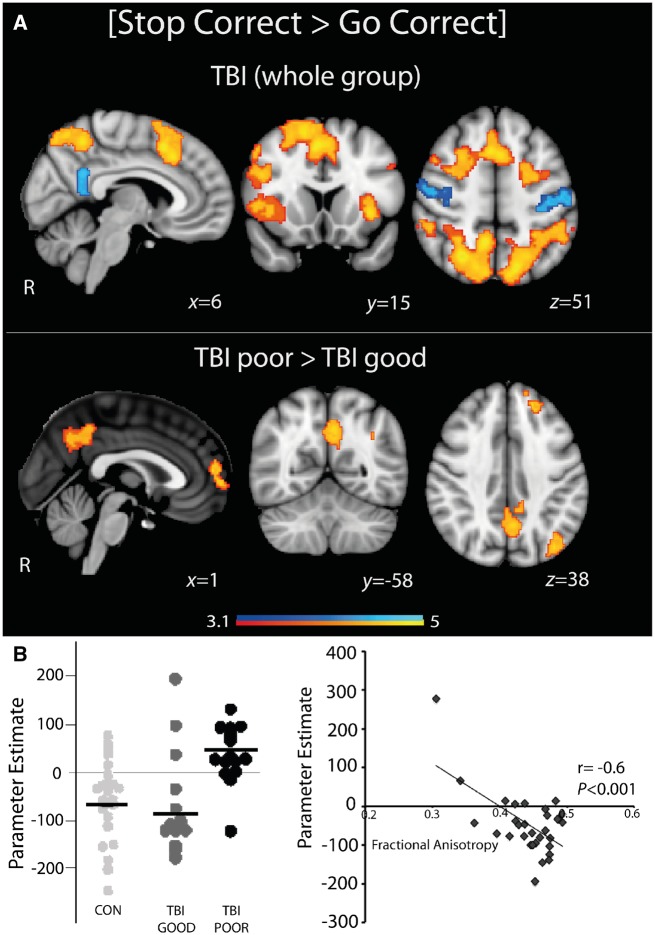
**Brain activation differences between good and poor TBI performers during successful stopping [Stop Correct > Go Correct].** (**A**) Overlay of areas of brain activation (warm colours) and deactivation (cool colours) during successful stopping for TBI participants. (**B**) Areas of greater brain activation during successful stopping in TBI poor performers compared to TBI good performers. Results are superimposed on the MNI152 1 mm brain template. Cluster corrected z = 3.1, *P < *0.05. *Left*: Graph shows activation from regions illustrated, black lines are group mean values. *Right*: Relationship between individual rAI-dACC/pre-SMA tract fractional anisotropy and default mode network BOLD response values during successful stopping.

During successful stopping TBI and control participants showed activation in parts of the salience network, including the dACC/pre-SMA and right anterior insular cortex (rAI), as well as bilateral superior frontal and superior parietal cortices. Deactivation was observed in bilateral primary motor and the posterior cingulate cortex (PCC), the central node of the default mode network (Fig. 4A). Replicating our previous results, poor TBI performers show abnormal default mode network activation in the posterior cingulate cortex and medial prefrontal cortex, while good TBI performers and controls showed a normal pattern of default mode network deactivation (Fig. 4B). Furthermore, as previously, structural connectivity of the rAI-dACC/pre-SMA tract was negatively correlated with default mode network activation across all TBI patients (r = −0.6, *P < *0.001) (Fig. 4B, right) (Bonnelle *et al.*, 2012), i.e. TBI patients with higher fractional anisotropy of the rAI-dACC/pre-SMA tract showed greater deactivation of the default mode network. These effects were specific to TBI patients, as poor control performers showed increased activation in sensorimotor areas, and dACC areas (Supplementary Fig. 4). However, there were no whole-brain activation differences on direct comparison of the whole TBI group with controls.

#### Anodal TDCS modulates abnormal default mode network activation patterns in poor TBI performers

**Figure 5:**
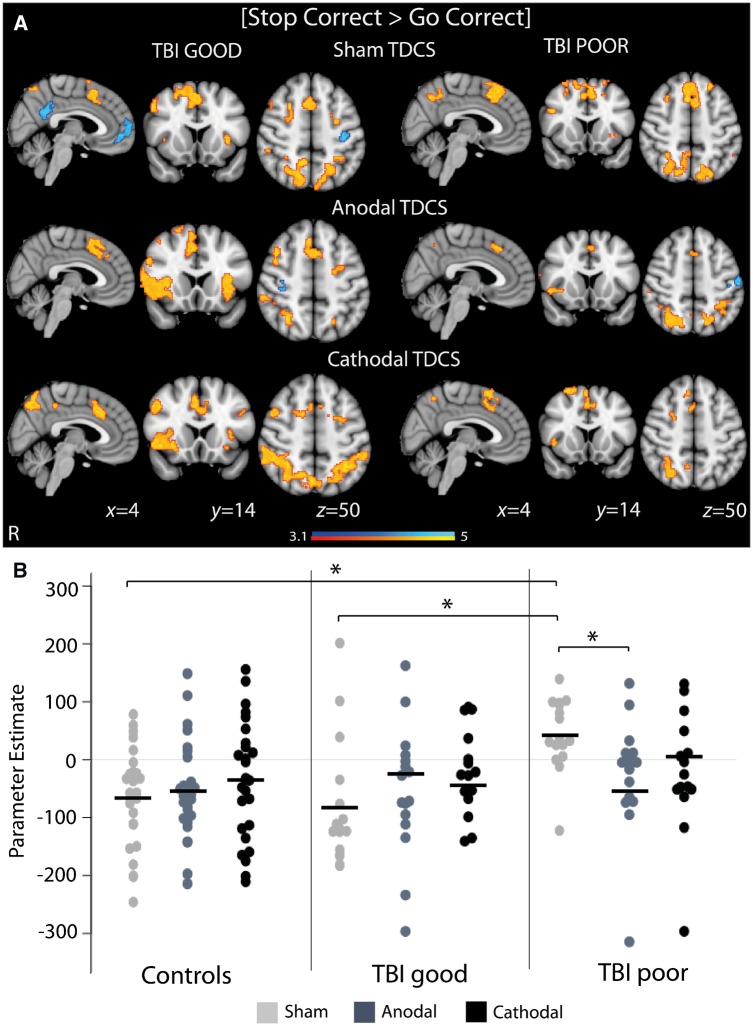
**Brain network response to TDCS.** (**A**) Overlay of areas of brain activation (warm colours) and deactivation (cool colours) during successful stopping for good and poor TBI performers under sham, anodal and cathodal TDCS. Results are superimposed on the MNI152 1 mm brain template. Cluster corrected z = 3.1, *P < *0.05. (**B**) Activity within the default mode network for good and poor TBI performers and controls under sham, anodal and cathodal TDCS (region of interest for extracting parameter estimates comprised the binarized mask of the voxelwise result presented in Fig. 4A, *bottom panel*). Black lines are group mean values. *P < 0.05.

During successful stopping, anodal and cathodal TDCS showed largely preserved patterns of activation, compared to sham stimulation, in both good and poor TBI performers (Fig. 5A). Distinct effects of stimulation were seen in brain regions where poor TBI performers had abnormally high activation (Fig. 5B). Compared to sham, anodal TDCS reduced activity during successful stopping within the default mode network. That is, abnormally high default mode network activity was significantly reduced by anodal TDCS, such that default mode network activity was no longer different from good TBI performers or controls. Stimulation showed no effect on default mode network activity in either good TBI performers or controls (Fig. 5B). There was no significant effect of cathodal TDCS or correlation between the TDCS-induced changes in blood oxygen level-dependent activity and task performance.

### Neuroimaging: network connectivity

#### Poor TBI performers had lower functional connectivity between the salience and default mode networks

**Figure 6:**
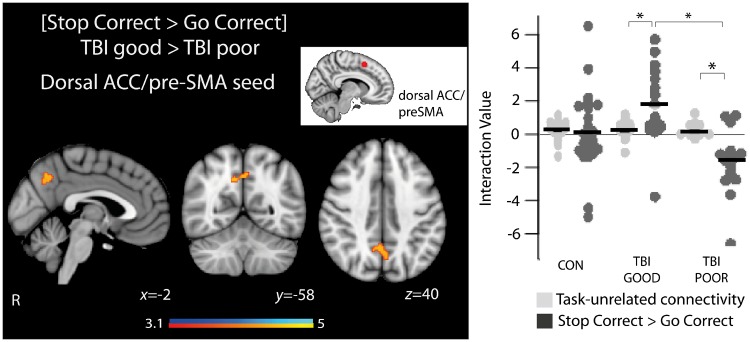
**Functional connectivity differences between good and poor TBI performers.** Overlay of areas of brain activation where functional connectivity within the dACC/pre-SMA motor node of the salience network is greater in good TBI performers than poor TBI performers. The accompanying graph shows the interaction values between the overlaid regions and the seed region for task-unrelated connectivity (pale grey) and for [Stop Correct > Go Correct] (dark grey). Black lines are group mean values. *Inset* shows the region of interest used as the seed region for the PPI analysis. *P < 0.05.

We have shown previously that TBI patients with impaired SST performance do not show the normal increase in functional connectivity between the default mode network and the rAI of the salience network during successful stopping (Jilka *et al.*, 2014). Here, we observed a reduction in functional connectivity between the default mode network and the dACC/pre-SMA node of the salience network in poor TBI performers during successful stopping (Fig. 6). Good performers showed an increase in the functional connectivity between the dACC/pre-SMA and the precuneus during successful stopping, whereas poor TBI performers showed a decrease in functional connectivity. This difference in connectivity was not observed when comparing good and poor control performers. There was no difference between good and poor TBI performers in the functional connectivity of the three default mode network nodes we investigated. There was no effect of stimulation on the functional connectivity.

### Predictive modelling

We performed an exploratory analysis to investigate the extent to which a wider range of predictors related to participants’ demographic variables, injury and structural and functional MRI characteristics might explain the variability in response to TDCS using linear mixed modelling.

#### White matter structure influences behavioural response to TDCS across all participants

Initially we assessed the following factors: age, stimulation type, rAI-dACC/pre-SMA tract fractional anisotropy, default mode network activation during successful stopping, and rAI-default mode network functional connectivity during successful stopping. Using a backward step-wise approach, we removed factors not significantly contributing to explaining the response to TDCS. The final combined model included the following variables: stimulation type and the fractional anisotropy of the rAI-dACC/pre-SMA tract, and explained 70.1% of the variability in response to TDCS. There was a main effect of rAI-dACC/pre-SMA tract fractional anisotropy on ΔSSRT [*F*(1,51) = 9.68, *P = *0.003]. There was no interaction between stimulation type and rAI-dACC/pre-SMA tract fractional anisotropy [*F*(1,51) = 1.59, *P = *0.119].

#### Time since injury is predictive of response to TDCS in patients

In a separate model, investigating factors that might explain variability of response to TDCS in TBI patients only, we additionally assessed: cingulum fractional anisotropy, time since injury, lesion volume % and whether patients had a significantly impaired MNC score. The final patient-only model included the following variables: rAI-dACC/pre-SMA tract fractional anisotropy, cingulum tract fractional anisotropy and time since injury, and explained 74.9% of the variability in response to TDCS. There was a main effect of time since injury on ΔSSRT [*F*(1,27) = 12.18, *P = *0.002], with a longer time since injury being associated with a bigger improvement in SST performance with TDCS. *Post hoc* correlations showed that time since injury predicted behavioural response to both anodal and cathodal TDCS (dSSRT_anodal_: r = −0.41, *P = *0.020; dSSRT_cathodal_: r = −0.42, *P = *0.019). Within this model, there was no main effect of cingulum or rAI-dACC/pre-SMA tract fractional anisotropy on ΔSSRT [default mode network: *F*(1,27) = 3.27, *P = *0.082; salience network: *F*(1,27) = 1.19, *P = *0.284]. There were no significant correlations between time since injury and rAI-dACC/pre-SMA or cingulum tract fractional anisotropy (rAI-dACC/pre-SMA tract r = 0.01, *P = *0.941; cingulum tract r = 0.12, *P = *0.507), nor any interaction between stimulation type and time since injury [*F*(1,29) = 0.886, *P = *0.383].

#### Perception of stimulation

Participants were asked after each run whether they thought they had real or sham TDCS. Their accuracy rates were consistent with chance, and there were no differences between stimulation conditions in ratings of perceived sensations.

## Discussion

We show that the behavioural response to brain stimulation after TBI is strongly related to the structure of the brain network stimulated. We used a novel simultaneous TDCS-MRI protocol and targeted the right inferior frontal gyrus/anterior insula (rIFG/AI). This is a key node within the salience network, which we have previously shown to produce executive dysfunction when damaged by TBI (Bonnelle *et al.*, 2012; Jilka *et al.*, 2014). Anodal TDCS improved response inhibition in healthy subjects, who have intact salience network structural connectivity. These benefits were not observed across the whole TBI group. Rather, the behavioural effects of TDCS correlated with the integrity of the tract connecting the rIFG/AI to medial frontal regions; we have previously shown that damage in this tract produces impairments of response inhibition (Bonnelle *et al.*, 2012; Jilka *et al.*, 2014). The cognitive effects of anodal TDCS on response inhibition inversely correlated with damage in this tract i.e. anodal stimulation produced the greatest behavioural improvements in TBI patients with the least amount of white matter damage. In addition, anodal stimulation normalized the function of the default mode network, a key marker of post-traumatic network dysfunction (Bonnelle *et al.*, 2012).

Our findings show that the integrity of white matter connections within a stimulated network is an important factor in predicting the response to non-invasive brain stimulation. This may reflect the importance of a network’s structural connectivity for its function. Variability in the strength of brain network structural connections influences communication across the network (Boorman *et al.*, 2007; Honey *et al.*, 2009; Horn *et al.*, 2014). Damage to these white matter connections impairs network communication and produces functional impairments (Mesulam, 1990; Kinnunen *et al.*, 2011). This is particularly important following TBI because traumatic axonal injury often disrupts structural connectivity due to axonal loss, which may occur at the time of injury through axonal shearing or in the chronic period with inflammation, delayed axonal degeneration and demyelination (Johnson *et al.*, 2013; Hill *et al.*, 2016). We have shown previously that post-traumatic damage to the structural connectivity of the rIFG/AI is associated with impaired network communication and executive dysfunction (Bonnelle *et al.*, 2012; Jilka *et al.*, 2014). Hence, the effect of stimulating the rIFG/AI may be influenced by similar post-traumatic damage to its connections within the salience network. In an extreme case, the complete disruption of the connections of this region would prevent the node from exerting any influence on the rest of the network and presumably remove any beneficial effects of its stimulation. With less severe tract disruption, the effects of stimulation would be reduced because of less efficient communication between nodes in the network.

A related mechanism underlying the influence of structural connectivity on the response to brain stimulation is through an effect of TDCS on axonal polarization i.e. a direct effect of the stimulation on axonal membrane potentials rather than via effects at the soma. Whilst most work has assumed a main effect of TDCS at the cortex, recent evidence shows that polarization effects can be stronger at axon terminals (Chakraborty *et al.*, 2017). Our modelling predicts peaks of electric field strength in the salience network white matter tracts, supporting a potential effect at this location. Traumatic axonal injury might be expected to reduce the polarization caused by stimulation due to damage to internal axonal structure and demyelination. This would potentially reduce the effects of stimulation, a hypothesis that could be tested directly by targeting stimulation directly to white matter tracts.

Another possibility is that the electric field strength distribution produced by stimulation is modulated by the structure of underlying tracts. Reductions of fractional anisotropy were seen in the white matter tracts we studied, a change that reflects axonal damage and altered fibre orientations within a tract (Winston, 2012). Modelling studies have shown that increasing the white matter fractional anisotropy results in more focused peaks of cortical stimulation, possibly because a higher fractional anisotropy reflects more consistent fibre orientation and reduces the spread of current away from the cortex (Metwally *et al.*, 2012; Shahid *et al.*, 2012; 2014). This effect of white matter fractional anisotropy on current distribution is thought to be relatively minor in healthy controls (Shahid *et al.*, 2014), but may be a larger influence in a TBI population, in whom axonal injury may result in widespread but heterogeneous disruptions in axonal orientation (Johnson *et al.*, 2013). In addition, an increased CSF volume due to lesions close to the stimulating electrode can lead to enhanced current shunting, resulting in reduced electric fields (Opitz *et al.*, 2015). As an extreme example, a patient with a large lesion directly under the cranial electrode would be expected to have little current reaching brain tissue. One previous study modelled the effect of cortical and subcortical parietal lesions in three stroke patients (Minjoli *et al.*, 2017), and found that lesions did not significantly affect the electric field strength distribution, but reduced the average peak values achieved. However, the lesions in this study were large, and close to the site of the stimulating electrode. Additionally, exclusion of participants with lesions overlapping any part of the rIFG, rAI or rAI-dACC/pre-SMA track did not alter the relationship between the behavioural response to anodal stimulation and rAI-dACC/pre-SMA tract fractional anisotropy. Therefore, trauma-induced change in the brain’s conductivity is unlikely to be the main mechanism for this finding

Two previous studies in stroke patients have demonstrated a relationship between white matter integrity after injury and the response to TDCS. First, a higher volume of the tract linking the right Broca’s area and the pre-SMA was associated with improved picture naming after cathodal TDCS was applied to right Broca’s area (Rosso *et al.*, 2014). Second, higher fractional anisotropy within the corticospinal tract was associated with improved muscle excitability of the paretic upper arm after contralesional cathodal TDCS (Bradnam *et al.*, 2012). Our study extends this work by investigating TBI patients for the first time and showing that the relationship between post-injury white matter integrity and behavioural gains is also relevant for cognitive function. Furthermore, we show that this relationship is specific to the integrity of connections in the stimulated network, since indicators of general brain tissue damage, such as lesion volume and the mean fractional anisotropy of the whole white matter skeleton, were not correlated with behavioural response to anodal TDCS.

We also observed that increasing time since TBI correlated with an improved behavioural response to anodal stimulation. This is in line with findings from studies of TDCS for motor deficits after stroke, where an increasing time since stroke was associated with greater behavioural gains from TDCS (Marquez *et al.*, 2013; O’Shea *et al.*, 2014). These observations are important as they suggest that TDCS may be most effective as a treatment in the chronic period. We did not observe a correlation between time since injury and measures of axonal injury, indicating that the influence of time since injury on response to TDCS does not simply reflect change in white matter integrity over time. Our results suggest that future studies of TDCS should explicitly evaluate how the timing of intervention relates to their efficacy.

The default mode network is a key network involved in cognitive function (Buckner *et al.*, 2008; Leech and Sharp, 2014). Previously, we have used functional MRI to investigate how TBI affects the default mode network and how this relates to cognitive impairment (Bonnelle *et al.*, 2011; Sharp *et al.*, 2011; Jilka *et al.*, 2014). Recording functional MRI simultaneous to delivering TDCS allowed us to study the effects of stimulation on default mode network function. We replicate our observations that abnormal default mode network activation and communication between the salience and default mode networks are seen in TBI patients with poor cognitive control, and that these abnormalities of network function are associated with white matter damage in the salience network (Bonnelle *et al.*, 2012; Jilka *et al.*, 2014). We extend these findings by showing that anodal TDCS ameliorated the abnormal default mode network activation in the poor TBI performers. These findings support the importance of the salience network–default mode network functional connectivity to cognitive control and show that TDCS applied to a remote but connected network provides a way to normalize network dysfunction after TBI. An interesting extension to this study would be to investigate whether direct modulation of the default mode network can ameliorate post-TBI cognitive dysfunction.

We did not find a correlation between TDCS-related changes in default mode network activation and behaviour, despite the finding that abnormal default mode network activation is seen in TBI patients in poor cognitive control. It is possible that abnormal default mode network activation is a biomarker of the network dysfunction, which contributes to poor cognitive control after TBI, rather than directly causal, making it a poor indicator of treatment effects. Another possibility is that normalization of default mode network activation is only one of the network changes that lead to behavioural changes. A final possibility, which fits with our finding that axonal damage in the salience network is related to stimulation response, is that stimulation-induced normalization of default mode network activation cannot lead to behavioural improvements in the presence of significant axonal damage in the salience network. Using multimodal techniques in future studies may help discover measures which are good biomarkers for both disease and treatment efficacy.

Our study has a number of limitations. As a group, TBI patients were not significantly impaired at baseline compared to control participants on the SST. Heterogeneity of recovery is commonly observed, and the baseline performance of our TBI participants was highly variable, following a bimodal distribution. This highlights the need to specifically account for how sources of heterogeneity might influence response to any intervention (Maas *et al.*, 2017). A second limitation is that we only stimulated a single node of the salience network, so it is not possible to conclude whether the effects observed reflect properties of the rIFG/AI specifically or the salience network more generally. An extension to help answer this question would be to stimulate the other node of the salience network, possibly using methods with deeper penetrance, such as temporal interference (Grossman *et al.*, 2017). A possible limitation is that we did not explicitly correct for haemosiderin staining or lesion volume in our analysis of DTI. However, lesion volume % did not correlate with either DTI metrics or behavioural effects of stimulation. Additionally, excluding the patients who had small areas of haemosiderin or lesion overlapping the rIFG, rAI or rAI-dACC/pre-SMA tract did not impact the observed correlation between rAI-dACC/pre-SMA fractional anisotropy and behavioural response to anodal stimulation. Therefore, we do not think these are likely to be significant confounds in our findings. A further limitation is that the linear model we report should be validated in a separate cohort. We report an exploratory analysis as a first step to understanding the relative influence of a range of factors influencing the response to stimulation, such as white matter integrity, lesion volume, and patient age. This supports the importance of salience network white matter integrity as a modulator of TDCS efficacy, but this finding will need to be confirmed in future work.

In summary, we applied TDCS to the rIFG/AI node of the salience network to investigate the treatment of cognitive impairment after TBI. Our key finding is that the behavioural response to anodal TDCS is strongly influenced by white matter integrity of the stimulated network. This principle is likely to be relevant to a range of neurological and psychiatric conditions where white matter abnormalities are observed, including stroke and major depression (Marquez *et al.*, 2013; Lefaucheur *et al.*, 2017). Individual variability in the response to stimulation is a major barrier to clinical translation across a range of conditions. Therefore, our findings will improve the development of TDCS as a treatment modality, by highlighting the need to understand how the effects of stimulation interact with the structure of stimulated networks.

## Supplementary Material

awz252_Supplementary_Data

## Abbreviations

dACC: dorsal anterior cingulate
pre-SMA: pre-supplementary motor area
rIFG/AI: right inferior frontal/anterior insula node
SSRT: stop signal reaction time
SST: Stop Signal Task
TBI: traumatic brain injury
TDCS: transcranial direct current stimulation
