# Supplementary material for: Traumatic axonal injury influences the cognitive effect of non-invasive brain stimulation
Source: Brain. 2019 Aug 30;142(10):3280–93. doi: 10.1093/brain/awz252 (PMC6794939; doi:10.1093/brain/awz252)
Supplement: awz252_Supplementary_Data [file awz252_supplementary_data.pdf]

SUPPLEMENTARY INFORMATION: METHODS

Figure 1: Exclusion of participant with lesions within the Salience Network

One TBI participant was found to have extensive white matter damage in the Salience Network (SN), despite intact overlying cortex (*Figure 1A*). He also had a very low FA value within the salience network white matter tract (connecting the right anterior insula to the dorsal anterior cingulate cortex/pre-supplementary motor area), >2.5SD below the group mean. Including his data did not make a difference to the significance of the relationship between Salience Network tract FA and DMN activation during successful response inhibition. Therefore, we excluded his data from further structural connectivity analyses.

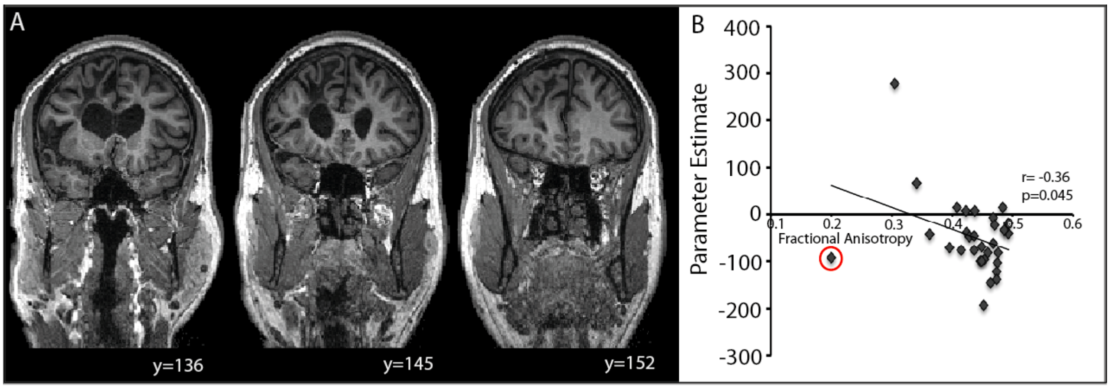

Figure 1: Participants relationship between white matter integrity and DMN activation

(A) T1 images for excluded participant showing extensive damage in the white matter connecting right inferior frontal to the dorsal anterior cingulate cortex/pre-supplementary area. (B) Relationship between salience network tract integrity and DMN activity during response inhibition with inclusion of this participant. Circled value is excluded TBI participant.

Table 1: participant characteristics summary

	TBI PARTCIPANTS	CONTROL PARTICIPANTS
Number	35	24
Age (years)	39.6 ± 10.1	39 ± 15.8
Gender	5F:30M	12F:12M
Time since injury (months)	48.9 ± 95.6	n/a
Impaired against controls based on MNC	19	n/a
Lesion Volume (%)	0.6 ± 1.3	n/a

Table 1: Characteristics of the TBI and control participant groups. Numbers are mean values ± standard deviation. F=female, M=male.

Transcranial Direct Current Stimulation

Anodal and cathodal TDCS was delivered with a ramp of 30s up to 2mA, followed by full intensity stimulation for the duration of the fMRI run, finishing with a ramp down over 1s. Sham TDCS consisted of the ramp stage only. Electrodes had a layer of conductive paste (Ten20, D.O. Weaver, Aurora, CO, USA), which held them in place and reduced impedances. Pre-stimulation impedances were below 3kΩ and maximum impedance during stimulation was 18kΩ. Heart rate was monitored concurrently using the pulse oximetry of the integrated Siemens Physiological Monitoring Unit. The set up and subsequent signal analysis has been previously described (Violante et al. 2017). There was no effect of stimulation on mean heart rate or its standard deviation.

After each run of TDCS-fMRI, participants were asked if they thought they had stimulation or not, and to rate (from 1-5) the sensations (itching, pain, metallic taste, burning, anxiety, anything else) they felt during the run.

Modelling of the current density

A computational model was used to confirm that our montage, with the use of an extracranial return, produced a peak electric field strength was over the rIFG. A finite element method (FEM) head model was created using Simnibs (Windhoff et al. 2013; Thielscher et al. 2015). This standard five compartment head model (WM, GM, CSF, skull and skin) was further extended to include neck and shoulder parts. Conductivity values for various tissues were used as in (Opitz et al. 2015). The electrode montage was modelled as described in the experimental section. Simulations of the tDCS electric field were performed using Simnibs v2.0.1. We did not use individual participant head models because the field of the view of the T1 scan precludes individual modelling of the shoulder return electrode, and because of the technical challenges involved in modelling lesions in injured brain (Minjoli et al. 2017). Given that tDCS electrodes were not directly placed above large lesions, the expected influence on the electrode field is likely limited.

Non-diffusion structural MRI acquisition & lesion analysis

T1-weighted high-resolution MPRAGE scans were acquired with 160 1-mm thick sagittal slices, repetition time=2000ms, echo time=2.98ms, flip angle=9°, in-plane resolution=1×1mm, matrix size=256×256, field of view=256×256mm. Fluid Attenuated Inversion Recovery (FLAIR) scans were acquired with 160 1mm thick sagittal slices, TE=395ms, TR=5000ms, TI=1800ms, matrix=256x256, field of view=250x250mm, GRAPPA=2. Susceptibility Weighted Imaging (SWI) was acquired with 144 0.9mm thick transverse slices, TE=20.0ms, TR=28ms, flip angle=15°, matrix=256x256, field of view=230x180mm, GRAPPA=2. Structural MRIs were reviewed and reported by an experienced consultant neuroradiologist.

Lesions visible on T1 and/or FLAIR were manually segmented for assessment with the Interactive Image Segmentation Tool v1.8 (ImSeg) Software (Ben Glocker), and manually checked for overlap with the right inferior frontal gyrus, right anterior insula and the primary tract of interest (rAI-dACC/preSMA tract). The ANTS registration software package (http://stnava.github.io/ANTs/) (Avants et al. 2014) was used to register SWI images in to MNI space for manual assessment of overlap between haemosiderin and the rAI-dACC/preSMA tract.

FMRI acquisition & analysis

FMRI images were obtained using a T2*-weighted gradient-echo, echoplanar imaging (EPI) sequence, 3mm3 isotropic voxel, repetition time (TR) 2 s, echo time (TE) 30 ms, flip angle (FA) 80°, field of view 192 × 192 x 105 mm, 35 slices, GRAPPA acceleration factor = 2. Standard T1-weighted structural images were also acquired using an MP-RAGE sequence, 1 mm3 isotropic voxel, TR 2.3 s, TE 2.98 ms, inversion time 900 ms, FA 9°, field of view 256 × 256 mm, 256 × 256 matrix, 160 slices, GRAPPA acceleration factor = 2.

FMRI was acquired whilst participants performed the Stop Signal Task. Participants were presented with left and right pointing arrows (the go signal) to which they responded by pressing a button with their left or right index finger respectively. In 20% of trials, a red dot (the stop signal) appeared above the arrow after a variable time (the stop signal delay). Participants were instructed to withhold the prepared response if the stop signal appeared. A 'feedback' screen, showing "Speed up!", was presented if participants showed evidence of strategic slowing (Bonnelle et al. 2012). The entire task was 4mins 12secs long, consisting of 184 trials. The task was programmed in Matlab (Mathworks, Natick, MA) using Psychtoolbox (Brainard, 1997) and responses were recorded through a fiberoptic response box (NordicNeuroLab, Norway), interfaced with the stimulus presentation PC.

FMRI pre-processing:

Data pre-processing was performed using the FMRI Expert Analysis Tool (FEAT) Version 6.00, from FMRIB's Software Library (FSL (Smith et al. 2004; Jenkinson et al. 2012)). We performed motion correction using MCFLIRT (Jenkinson et al. 2002), removal of low-frequency drifts (high-pass filter of 0.01 Hz), spatial smoothing (Gaussian kernel filter with a full width at half maximum of 6 mm), brain extraction to remove non-brain tissue (BET (Smith 2002)), and co-registration using FMRIB's Nonlinear Image Registration tool (FNIRT) to register the participant's fMRI volumes to Montreal Neurological Institute (MNI) 152 standard space using the T1-weighted scan as an intermediate.

Single-session ICA was performed for each run using Multivariate Exploratory Linear Optimized Decomposition (MELODIC (Beckmann et al. 2005)). The resulting components were automatically classified into signal and noise using FMRIB's ICA-based Xnoiseifier (FIX (Griffanti et al. 2015; Salimi-khorshidi et al. 2015)). FIX was previously trained in an independent cohort of twenty individuals acquired in the same scanner with the same imaging parameters. Classifications were manually inspected and adjusted when required. Independent components classified as noise were subsequently removed from each voxel's time series.

fMRI analysis: activation

All analyses were performed with FSL's FMRI Expert Analysis Tool (FEAT). Subject-level general linear models (GLM) included 5 regressors of interest from the task: Go Correct (trials in which correct responses were made to the go signal), Go Incorrect (trials in which incorrect responses were made to the go signal), Stop Correct (trials in which participants successfully withheld a response to the stop signal), Stop Incorrect (trials in which participants pressed a button despite the presentation of a stop signal) and Feedback (presentations of the feedback screen).

The GLM design matrix consisted of those regressors of interest, their first temporal derivatives and six movement regressors to account for movement-related noise. The following contrasts of interest were investigated [Stop Correct>Go Correct] and [Stop Correct>Stop Incorrect], and the inverse contrasts were also run.

fMRI analysis: functional connectivity

Functional connectivity between specific regions of interest (ROIs) and the rest of the brain was investigated with whole-brain PPI (O'Reilly et al. 2012). A separate group of healthy volunteers (n= 22, 4F:18M) (mean age 40 years, s.d. 12.4 years) performed a run of the Stop Signal Task with no TDCS circuit attached. The data from this group was preprocessed and analysed in the same way as the TDCS-fMRI cohort, and peaks of activation and deactivation were used to define the ROIs for the functional connectivity analyses of the TDCS-fMRI cohort. These ROIs were located in the Salience and Default Mode Networks.

The regions of interest were defined as follows:

- Right inferior frontal gyrus (rIFG): this was generated using MANGO (Multi-image Analysis GUI) software (http://ric.uthscsa.edu/mango/mango.html) by defining a 22.5 mm radius sphere centered on the centre coordinates F8. This was obtained from the projection of the electrode position onto the cortical surface (Koessler et al. 2009) and converted to MNI space using the Nonlinear Yale MNI to Talairach Conversion Algorithm (Lacadie et al. 2009) (Lacadie et al., 2008) (F8: x=55, y=30, z= –1). The ROI was predominantly over the pars triangularis of the rIFG. Areas outside the cortex were removed by masking with an MNI brain mask from the FSL library.
- Right anterior insula (rAI): this was defined by generating a 5mm diameter sphere around the peak activation voxel in the anterior regions, for the contrast [Stop Correct > Go Correct], in a separate group of healthy controls (see 'Participants'). MNI coordinates: x40, y20, z0.
- Dorsal anterior cingulate cortex/pre-supplementary motor area (dACC/pre-SMA): this was defined by generating a 5mm diameter sphere around the peak activation voxel in the anterior regions, for the contrast [Stop Correct > Go Correct], in a separate group of healthy controls (see 'Participants'). MNI coordinates: x8, y14, z52.
- Ventromedial prefrontal cortex (vmPFC): this was defined by generating a 5mm diameter sphere around the peak of deactivation in the frontal DMN regions, for the contrast [Stop Correct > Go Correct], in a separate group of healthy controls (see 'Participants'). MNI coordinates: x-8, y46, z-10.
- Dorsal and ventral posterior cingulate cortex (PCC): these were defined by generating 5mm diameter spheres around the coordinates given by Leech et al. We selected two nodes for PCC ROIs, rather than a large anatomical PCC, because connectivity between the ventral PCC and dorsal PCC is differentially modulated during a cognitively demanding task, with the dorsal PCC showing increased integration with the rest of the DMN with increasing task difficulty (Leech et al. 2011).

A generalised PPI model was used (McLaren et al. 2012). Time-courses for each region were extracted for each participant (the physiological term). Regressors used were the same as for the initial fMRI analysis. For each model, the physiological term and the psychological terms [Stop Correct], [Stop Incorrect] and [Go Correct] were

used to create the PPI interaction terms, and the remaining regressors also included in the model.

DTI acquisition and analysis
Diffusion-weighted volumes were acquired using a 64-direction protocol (64 slices, in-plane resolution=2x2mm, slice thickness=2mm, field of view = 256x256cm, matrix size=128x128, TR=9500ms, TE=103ms, b-value=1000s/mm^2). Four non-diffusion weighted images were also acquired (b-value=0s/mm^2).

DTI data were corrected for head motion and eddy current distortions, using linear transformations to register these images to the b=0 image. A brain mask was generated by brain extracting the b=0 image (FSL Brain extraction tool (Smith 2002)). A tensor model was then fitted to the data using FMRIB's Diffusion Toolbox (FDT) in FSL, constrained by the brain mask. Applying this tensor model generated voxelwise individual participant fractional anisotropy (FA) maps. These maps were transformed into 1mm-resolution standard space using DTI-TK (Zhang et al. 2006). An initial group based template was generated through bootstrapping of the tensor-based maps together with the predefined IXI aging standard template (Zhang et al. 2010). Individual tensor-based images were then registered to the group template using diffeomorphic transformations.

Linear mixed effects model construction
Predictive factors were checked for colinearity, heteroscedasticity and normality. Non-normal data was transformed using a Tukey transformation before inclusion in the model. Models were constructed using a backward, step-wise approach. Models were compared and the simplest model was selected for the next comparison. Other model metrics were also used to determine the best model: BIC (information criterion) and predicted R squared values were used to give an indication of risk of overfitting, and the standard deviation of the residuals were used to give an indication of how well the model fit the data (Cheng et al. 2010).

The following factors were included in the initial maximal combined model:
- age
- FA within the rAI-dACC/preSMA tract
- DMN activation during response inhibition
- rAI-DMN functional connectivity during response inhibition

The following factors were additionally included in the patient-only model:
- FA within the cingulum (mPFC-PCC/PRE) – this is because there was an interaction between the FA and GROUP (TBI or control)
- time since injury (in months)
- lesion volume %
- whether patients had a significantly impaired MNC score

References
Avants, B.B. et al., 2014. The Insight ToolKit image registration framework. *Front Neuroinform,* 8(44).
Beckmann, C.F. et al., 2005. Investigations into resting-state connectivity using independent component analysis. *Philosophical Transactions of the Royal Society B*, 360(May), pp.1001–1013.

Bonnelle, V. et al., 2012. Salience network integrity predicts default mode network function after traumatic brain injury. *PNAS*, 109(12), pp.4690–4695.
Cheng, J. et al., 2010. Real longitudinal data analysis for real people: building a good enough mixed model. *Statistics in Medicine*, 29, pp.504–520.
Griffanti, L. et al., 2015. ICA-based artefact removal and accelerated fMRI acquisition for improved Resting State Network imaging. *Neuroimage*, 95, pp.232–247.
Jenkinson, M. et al., 2012. FSL. *Neuroimage*, 62, pp.782–790.
Jenkinson, M. et al., 2002. Improved Optimization for the Robust and Accurate Linear Registration and Motion Correction of Brain Images. *Neuroimage*, 841, pp.825–841.
Koessler, L. et al., 2009. Automated cortical projection of EEG sensors: Anatomical correlation via the international 10-10 system. *NeuroImage*, 46(1), pp.64–72.
Lacadie, C. et al., 2009. More Accurate Talairach Coordinates for NeuroImaging using Nonlinear Registration. *Neuroimage*, 42(2), pp.717–725.
Leech, R. et al., 2011. Fractionating the Default Mode Network : Distinct Contributions of the Ventral and Dorsal Posterior Cingulate Cortex to Cognitive Control. *J Neurosci*, 31(9), pp.3217–3224.
McLaren, D.G. et al., 2012. A generalized form of context-dependent psychophysiological interactions (gPPI): A comparison to standard approaches. *NeuroImage*, 61(4), pp.1277–1286.
O'Reilly, J.X. et al., 2012. Tools of the trade: Psychophysiological interactions and functional connectivity. *Soc Cogn Affect Neurosci*, 7(5), pp.604–609.
Opitz, A. et al., 2015. Determinants of the electric field during transcranial direct current stimulation. *NeuroImage*, 109, pp.140–150.
Salimi-khorshidi, G. et al., 2015. Automatic Denoising of Functional MRI Data: Combining Independent Component Analysis and Hierarchical Fusion of Classifiers. *NeuroImage*, 90(0), pp.449–468.
Smith, S.M. et al., 2004. Advances in Functional and Structural MR Image Analysis and Implementation as FSL Technical Report TR04SS2. *Neuroimage*, 23(S1), pp.208–219.
Smith, S.M., 2002. Fast Robust Automated Brain Extraction. *Hum Brain Mapp*, 155, pp.143–155.
Thielscher, A., Antunes, A. & Saturnino, G.B., 2015. Field modeling for transcranial magnetic stimulation: a useful tool to understand the physiological effects of TMS? *IEEE Trans Biomed Eng*, pp.222–225.
Violante, I.R. et al., 2017. Externally induced frontoparietal synchronization modulates network dynamics and enhances working memory performance. *eLife*, 6, pp.1–22.
Windhoff, M., Opitz, A. & Thielscher, A., 2013. Electric field calculations in brain stimulation based on finite elements: an optimized processing pipeline for the generation and usage of accurate individual head models. *Hum Brain Mapp*, 34(4), pp.923–935.
Zhang, H. et al., 2006. Deformable registration of diffusion tensor MR images with explicit orientation optimization. *Medical Image Analysis*, 10(5), pp.764–785.
Zhang, H. et al., 2010. The computational aging DTI template with support for tract-specific analysis. In *International Workshop on Biomedical Image Registration*. pp. 83–90.

SUPPLEMENTARY INFORMATION: RESULTS

Table 2: summary of TBI patient structural MRI images

Participant ID	Report Summary
TBI_01	Mature damage and haemosiderin staining in L SFG, L MFG, L inferior frontal lobe, anterior temporal pole and posterior aspect L frontal operculum, R parieto-occipital and posterior temporal regions. Microhaemorrhages in b/l frontal border zone, dorsal pons.
TBI_02	Mature parafalcine gliotic damage, L more than R. L temporal microhaemorrhages.
TBI_03	Mature contusions b/l frontal poles, L lateral MTG. Microhaemorrhages L perisylvian cortex.
TBI_05	Mature parafalcine contusions with L postcentral gyrus contusions, haemosiderin and damage of L thalamus. Subcortical microhaemorrhages bilaterally.
TBI_06	Wallerian degeneration in the corticspinal tract. Evidence of petechial microhaemorrhages particularly in the right temporal lobe, L medial temporal lobe and left dorsal lentiform nucleus.
TBI_07	Mature contusions with haemosiderin L frontal and parietal regions. Microhaemorrhages in R superior cerebellar pontine peduncle, R medial occipitotemporal gyrus, multiple perisylvian microhaemorrhages particularly on L.
TBI_08	Small area of superficial siderosis in R SFG.
TBI_09	Small contusion L occipital pole, some R pontine and L hemisphere atrophy.
TBI_11	No mature contusions or microhaemorrhages
TBI_12	R temporal contusions, R fronto-parietal-temporal superficial siderosis, parafalcine microhaemorrhages.
TBI_13	R frontal and temporal pole superficial haemosiderin staining, superficial siderosis staining inferior surface R cerebellar
TBI_14	Multiple subcortical microhaemorrhages, superficial siderosis R parietal and mature haemorrhagic contusions anterior frontal
TBI_15	Bifrontal mature contusions, signal change at R parieto-occipital fissure possibly consistent with shear injury.
TBI_16	R dorslolateral frontal and lateral temporal mature contusion

TBI_17	L temporal and inferior frontal contusional gliotic damage, L parietal microhaemorrhage, extensive L superficial siderosis
TBI_19	Tiny T2* lesions in R anterior MFG.
TBI_21	L frontal sclerotic damage with haemosiderin.
TBI_22	Non-specific subcortical WM spots.
TBI_23	B/L mature inferior frontal pole and temporal pole contusions, few microhaemorrhages R MTL, L temporal pole.
TBI_24	Few microhaemorrhages in L MTL.
TBI_25	Microhaemorrhages parafalcine (R mostly), superficial haemosiderin deposition.
TBI_26	Widespread microhaemorrhages.
TBI_27	Mature haemorrhagic contusions R occipitotemporal, L lateral temporal, L dorsolateral frontal. Subcortical microhaemorrhages, especially R occipitotemporal.
TBI_28	Scattered microhaemorrhages
TBI_29	Mature R temporal and subfrontal contusion, L frontal microhaemorrhage
TBI_30	Central sulcus superficial siderosis
TBI_31	Microhaemorrhages L frontal parafalcine, B/L temporal, brainstem. Mature gliotic contusion R occipital.
TBI_32	Microhaemorrhages L temporal pole, R SFG. Previous L cerebral peduncle haemorrhagic.
TBI_33	Multiple microhaemorrhages frontal and deep white matter. Frontal pole signal change.
TBI_34	Extensive microhaemorrhages R temporal, L cerebral peduncle. L SFG superficial siderosis. Perivenular haemorrhages R SFG and L MFG. Mature SFG & R supraorbital frontal contusions.
TBI_35	Widespread microhaemorrhages. Mature R frontal contusions.

Table 2: summary of TBI patient structural MRI images

Reports were based on T1, FLAIR and SWI/T2*GRE images. TBI_16 did not have SWI or T2*GRE images available. As described in 'Methods' of main manuscript, 4 participants were excluded (TBI_04 for large white matter lesion within the Salience

Network [see SI Figure 1]; TBI_10 for large cortical and subcortical lesion in right frontal region; TBI_18 and TBI_20 for failing Stop Signal Task performance criteria). Abbreviations: MFG=middle frontal gyrus, SFG=superior frontal gyrus, MTG=middle temporal gyrus, L=left, R=right, b/l=bilateral

Figure 2: distribution of baseline SST performance in TBI participants

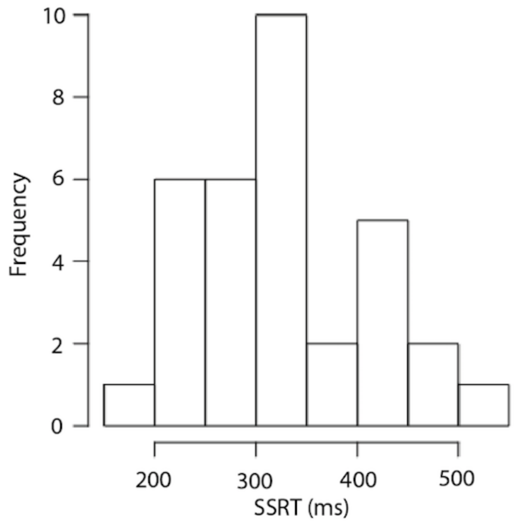

Figure 2: distribution of baseline SST performance in TBI participants
Histogram of SSRT (ms) from the sham condition in TBI participants

Table 3: behavioural measures for Stop Signal Task performance

	**Anodal**	**Cathodal**	**Sham**
CONTROLS			
SSRT (ms)	291.3 ± 58.4	295.1 ± 58.6	321.7 ± 48.0
Stop Signal Delay (ms)	253.7 ± 159.3	230.8 ± 105.7	200.8 ± 94.9
Incorrect stop RT (ms)	523.0 ± 104.7	495.8 ± 71.5	496.1 ± 75.1
Mean RT (ms)	545.1 ± 121.1	525.9 ± 87.6	522.6 ± 89.4
TBI			
SSRT (ms)	343.4 ± 73.0	326.5 ± 62.6	328.1 ± 79.8
Stop Signal Delay (ms)	216.9 ± 88.3	234.0 ± 95.0	235.1 ± 131.1
Incorrect stop RT (ms)	539.7 ± 78.5	544.1 ± 67.0	541.6 ± 77.0
Mean RT (ms)	575.1 ± 86.2	577.5 ± 76.8	578.6 ± 83.1

Table 3: Behavioural measures for the Stop Signal Task.

These values are the summary statistics for Figure 2 in the main manuscript. Values represent mean ± standard deviation. Abbreviations: SSRT = stop signal reaction time, RT = reaction time, ms = milliseconds

Figure 3: Relationship between behavioural response to cathodal stimulation ($\Delta SSRT_{cathodal}$) and white matter integrity

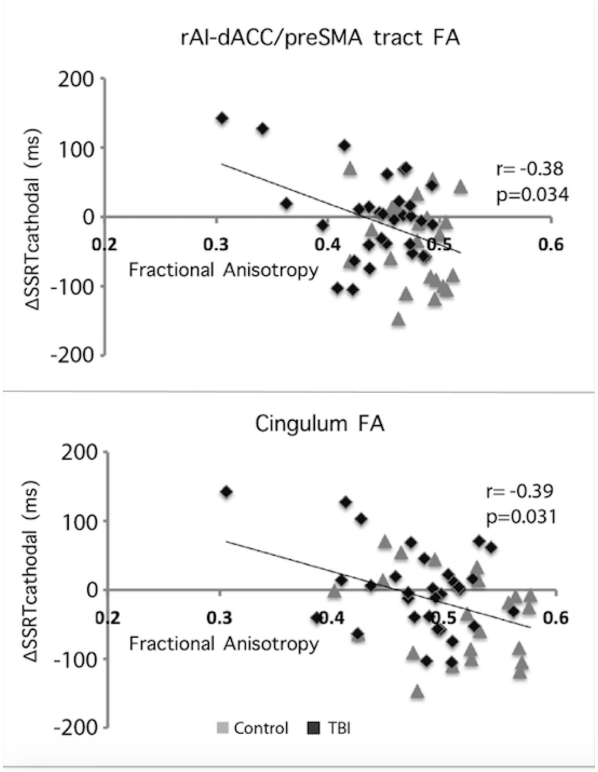

Figure 3: Relationship between behavioural response to cathodal stimulation and white matter tract integrity

Correlation between the $\Delta SSRT_{cathodal}$ and FA within the rAI-dACC/preSMA tract (top panel) and within the cingulum (bottom panel), across both control (light grey) and TBI (dark grey) participants.

Figure 4: Comparing good and poor control performers during successful stopping

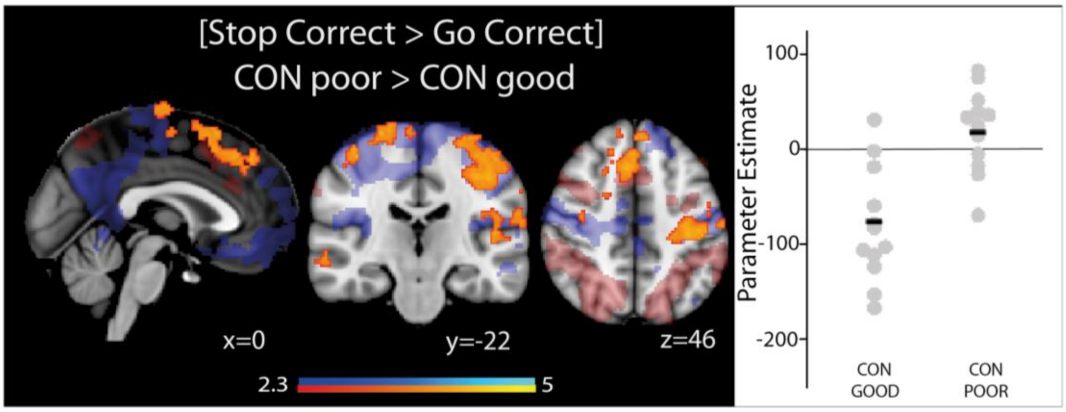

Figure 4: Comparing brain activation between good and poor control performers (based on median split of SSRT during the sham condition)

Overlay of areas of greater brain activation (warm colours) during successful stopping in poor control performers compared to good control performers. The translucent areas indicate areas of brain activation (red) and deactivation (blue) during successful stopping across the whole control group. The accompanying chart shows individual activations within these areas (black line denotes group mean). Results are superimposed on the MNI152 1mm brain template. Cluster corrected z=2.3, p<0.05.
